# Hybrid calcium carbonate/polymer microparticles containing silver nanoparticles as antibacterial agents

**DOI:** 10.1007/s11051-012-1313-7

**Published:** 2012-11-25

**Authors:** Maciej Długosz, Maria Bulwan, Gabriela Kania, Maria Nowakowska, Szczepan Zapotoczny

**Affiliations:** Faculty of Chemistry, Jagiellonian University, Ingardena 3, 30-060 Krakow, Poland

**Keywords:** Silver nanoparticles, Antimicrobial agent, Calcium carbonate microparticles, Controlled release

## Abstract

We report here on synthesis and characterization of novel hybrid material consisting of silver nanoparticles (nAgs) embedded in calcium carbonate microparticles (μ-CaCO_3_) serving as carriers for sustained release. nAgs are commonly used as antimicrobial agents in many commercial products (textiles, cosmetics, and drugs). Although they are considered to be safe, their interactions with human organisms are still not fully understood; therefore it is important to apply them with caution and limit their presence in the environment. The synthesis of the new material was based on the co-precipitation of CaCO_3_ and nAg in the presence of poly(sodium 4-styrenesulfonate). Such designed system enables sustained release of nAg to the environment. This hybrid colloidal material (nAg/μ-CaCO_3_) was characterized by microscopic and spectroscopic methods. The release of nAg from μ-CaCO_3_ microparticles was followed in water at various pH values. Microbiological tests confirmed the effectiveness of these microparticles as an antibacterial agent. Importantly, the material can be stored as a dry powder and subsequently re-suspended in water without the risk of losing its antimicrobial activity. nAg/μ-CaCO_3_ was applied here to insure bacteriostatic properties of down feathers that may significantly prolong their lifetime in typical applications. Such microparticles may be also used as, e.g., components of coatings and paints protecting various surfaces against microorganism colonization.

**Electronic supplementary material:**

The online version of this article (doi:10.1007/s11051-012-1313-7) contains supplementary material, which is available to authorized users.

## Introduction

Silver, in the form of nanoparticles (nAg) and ions, is a common antimicrobial agent. It interacts with cell components (e.g., DNA, RNA, and ribosomal units), deactivating them and effectively stopping microbial life processes. It has been observed that nAg can puncture cell membranes and penetrate deep into the cell interior. This, in combination with their slow dissolution and ability to release Ag^+^ ions, results in excellent antimicrobial activity even at nanomolar concentrations (Sondi and Salopek-Sondi 2004; Yamanaka et al. 2005; Feng et al. 2000; Lok et al. 2006). nAg are effective agents even against multidrug-resistant strains (Lara et al. 2009). These exceptional properties of nAg resulted in wide range of their practical applications. They have been applied as an antibiotic additive in the production of biomedical devices (Roe et al. 2008; Cao and Liu 2010), wound dressings (Nguyen et al. 2011; Tian et al. 2007), water (Lv et al. 2009; Jain and Pradeep 2005) and air (Yoon et al. 2008) filters, textiles (Tang et al. 2011; Lee et al. 2003), food packaging (Tankhiwale and Bajpai 2009), cosmetics (Kokura et al. 2010), and drugs (Shahverdi et al. 2007).

However, it has been shown that the growing number of application of nAg may lead to the build up of silver concentrations in the environment to such a level at which it can not only cause health problems in humans (Kim et al. 2011) but also may be harmful for various components of the environment (Lee et al. 2007) and may even affect the industrially relevant processes (Brar et al. 2010). Therefore, it is important to use nAg rationally and to control their release to the environment. The current paper presents our approach to this problem. We have proposed to embed nAg into the biologically inert calcium carbonate microparticle (μ-CaCO_3_) matrix (Lei et al. 2006). Several other attempts to immobilize nAg with polymers have been already proposed (Benetti et al. 2010). They include immobilization on glass and silica (Cai et al. 2001; Lv et al. 2008), on natural fibers (Tang et al. 2011; Lee et al. 2003), on hydroxyapatite (Nirmala et al. 2011), and even on the surface of steel (Chen et al. 2010). However, to the best of our knowledge, the colloidal system containing silver nanoparticles trapped inside calcium carbonate microparticles (nAg/μ-CaCO_3_) has not been fabricated so far. Such application of μ-CaCO_3_ microparticles is in line with their most common usage as sacrificial templates for fabrication of hollow polymeric microcapsules (Volodkin et al. 2004) carrying various organic molecules such as low molecular weight drugs (Tong et al. 2011) or proteins (Petrov et al. 2005). The approach proposed here should insure long-lasting antibacterial activity of the material due to the sustained release of silver on one hand and limit the contact of nAg with human body on the other. In addition, this material can be easily stored as a water dispersible powder and its surface charge can be readily modified for various applications. This new material may find applications as a component of coatings and paints protecting the surfaces against microorganism colonization.

## Materials and methods

### Materials

Poly(sodium 4-styrenesulfonate) (PSS, Aldrich, *M*_w_ ≈ 70,000 g/mol), poly(allylamine hydrochloride) (PAH, Aldrich, *M*_w_ ≈ 15,000 g/mol). Silver nitrate, trisodium citrate, calcium nitrate, sodium carbonate, acetone, sulfuric acid (98 %), nitric acid (65 %), hydrogen peroxide (30 %), ethylenediaminetetraacetic acid (EDTA), disodium phosphate, and citric acid were obtained from POCH Gliwice, Poland. Single-side polished silicon plates were obtained from the Institute of Electronic Materials Technology (Warsaw, Poland) and cleaned before the use in freshly prepared “piranha solution” (a mixture of 30 % solution of H_2_O_2_ and concentrated H_2_SO_4_ in 1:3 ratio) (Caution: “piranha solution” should be handled with extreme care!). Deionized water was used in all experiments. Buffers were prepared by mixing appropriate amounts of 0.1 M citric acid and 0.2 Na_2_HPO_4_.

### Procedures

#### Synthesis of nAg/μ-CaCO_3_ material and its surface modification

In the first step, nAg were obtained by modified Lee and Meisel method (1982). Shortly, 45 mg of silver nitrate was dissolved in 250 ml of water and then 5 ml of 1 % trisodium citrate was added. The reaction mixture was placed in an ultrasonic bath and heated to 75 °C for 60 min. In the second step, 20 ml of the obtained nAg colloid was simultaneously mixed with 50 ml of 0.03 M Ca(NO_3_) solution and 50 ml of 0.03 Na_2_CO_3_ with addition of PSS (4.8 g/l). The mixture was sonicated for 5 min at 25 °C. The obtained white colloid was washed with deionized water and centrifuged at 4,000 rpm for 5 min to remove excess of silver. The washing process was repeated three times and the obtained product was dried under vacuum.

For surface modification, nAg/μ-CaCO_3_ particles were dispersed in PAH solution (1 g/l in 0.1 M NaCl) and stirred for 15 min. They were subsequently centrifuged and washed with deionized water.

#### Characterization of nAg

UV–Vis spectrum of the obtained nAg suspension was acquired. The size of the nanoparticles was determined by atomic force microscopy (AFM, Picoforce, Bruker, USA) working in tapping mode. Standard silicon cantilevers (Bruker) with nominal spring constants equal to 40 N/m were used for the measurements. For that purpose, a silicon plate was first immersed in PAH solution (1 g/l in 0.1 M NaCl) and sonicated for 5 min. The plate was subsequently rinsed with deionized water and dried under stream of argon. A drop of the colloidal suspension of nAg was deposited on such prepared silicon support and left for drying before the imaging.

#### Characterization of nAg/μ-CaCO_3_ material

Scanning electron microscopy images were obtained for nAg/μ-CaCO_3_ using Hitachi S-4700 microscope with field emission. The samples were coated with sputtered gold layer before imaging.

To check if silver in the form of nanoparticles is embedded in μ-CaCO_3_, the matrix was dissolved in 0.2 M EDTA and the UV–Vis spectrum of the obtained solution was measured.

Total content of silver in nAg/μ-CaCO_3_ was measured by atomic absorption spectrometry (AAS) using PerkinElmer Aanalyst 300 instrument with flame atomizer. The samples were transferred into solution by dissolving in 0.12 M HNO_3_. The appropriate calibration procedure was performed using samples of AgNO_3_ in 0.12 M HNO_3_. The measurements were also performed for the starting nAg dispersion and supernatant collected after centrifugation of the reaction mixture.

#### Accelerated release of nAg from nAg/μ-CaCO_3_

The samples of nAg/μ-CaCO_3_ (440 mg) were dispersed in 8 ml of the appropriate buffer solutions with pH values equal to 5.4 and 6.8. They were vigorously stirred for a given period of time and then centrifuged. Supernatants were collected for AAS analyses and the next portions of fresh buffer solutions were added each time. The procedure was continued for 14 days.

#### Treatment of down feathers

The nAg/μ-CaCO_3_ were introduced to the feathers by their immersion in the 0.6 % dispersion for 30 min. Then the feathers were dried under a stream of argon. The samples were investigated by optical microscopy (Nikon Eclipse LV 100) and SEM (Hitachi S-4700 s microscope) to confirm the attachment of nAg/μ-CaCO_3_ to the feathers.

#### In vitro antimicrobial activity

To prove the antimicrobial activity of the obtained materials, the microbiological tests were carried out. Various bacterial species were placed in physiological salt media to which equal amounts of nAg/μ-CaCO_3_ dispersion were added and left for 24 h. The bacterial and fungal species were naturally occurring skin microbes and were acquired from several healthy people. The microbial concentration was expressed in McFarland scale (°McF) based on the nephelometric measurements. One McFarland scale is equivalent to turbidity of standard BaSO_4_ colloid in concentration of 4.80 × 10^−5^ M. nAg/μ-CaCO_3_ as well as down/nAg/μ-CaCO_3_ and sole nAg dispersion systems were tested. The initial turbidity was acquired before the addition of the antimicrobial materials or down feathers. The concentration of the antimicrobial hybrid material was kept constant in each test and it was allowed to sediment before the measurements of the microbial concentration. The feathers, after the incubation, were removed from the dispersion. In the case of sole nAg dispersion, the total turbidity (with contribution of nAg) was measured. The respective microbial concentrations were taken as mean values of a few measurements.

## Results and discussion

Successful two-step synthesis of novel antimicrobial material consisting of nAg embedded in μ-CaCO_3_ microparticle matrix was achieved. The nAg were obtained by modified method developed earlier by Lee and Meisel. The method is based on the reduction of silver nitrate by sodium citrate in hot water (Lee and Meisel 1982). It is important to note that the process is carried out in aqueous medium and with the use of low toxicity reagents. Citrate ions play also a second function—they protect Ag nanoparticles form aggregation, thus keeping their size relatively small. This is very important considering the fact that the antimicrobial activity of nAg rises significantly with the reduction of their sizes (Panáček et al. 2006).

**Fig. 1:**
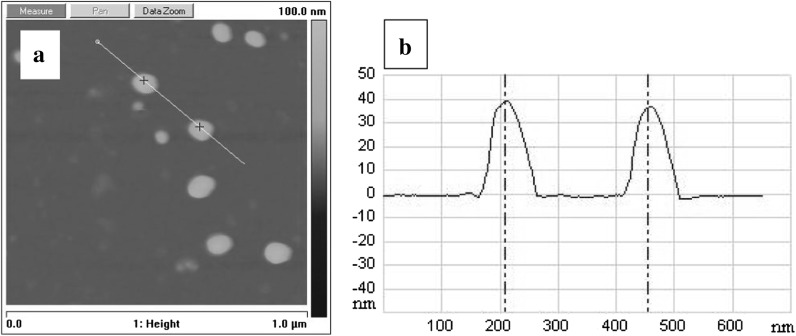
**a** AFM image and **b** the corresponding cross section of the obtained nAg adsorbed on positively charged layer of PAH on silicon

**Fig. 2:**
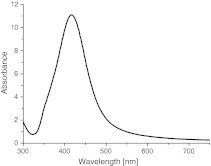
UV–Vis absorption spectrum of the obtained nAg suspension

In a current study, the method was modified by the application of ultrasonic waves and lowering the reaction temperature to 75 °C which resulted in formation of fine (30–50 nm) and more monodisperse nAg as shown by AFM (Fig. 1). The surface charge of the synthesized nAg is negative due to the presence of citrate ions adsorbed. This was confirmed by the observation of strong adsorption of nAg on PAH covered silica plate characterized by positive surface charge (Fig. 1) and lack of adsorption on negatively charged, unmodified silica plates. The obtained colloidal suspension was also characterized spectroscopically. Green color of nAg colloid and its characteristic UV–Vis spectrum with the maximum around 420 nm (Fig. 2) originates from localized surface plasmon resonance of these metallic nanostructures (Kelly et al. 2002).

**Fig. 3:**
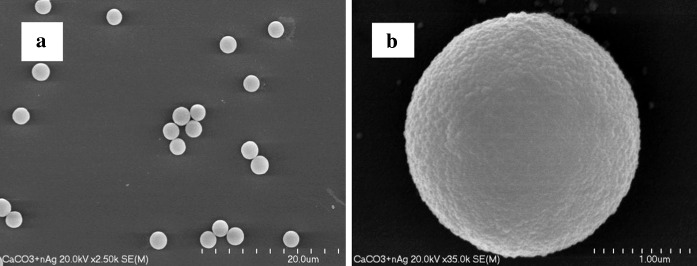
SEM images of nAg/μ-CaCO_3_ at different magnifications

**Fig. 4:**
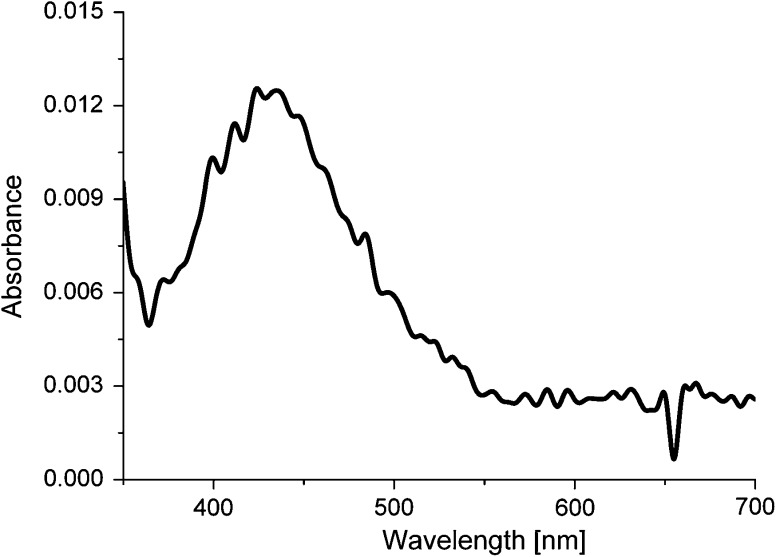
UV–Vis spectrum of nAg/μ-CaCO_3_ dispersion after the dissolution of the carbonate matrix using EDTA

The obtained nanoparticles were then embedded into μ-CaCO_3_ matrix by co-precipitation in the presence of the polyelectrolyte (PSS) resulting in the formation of nAg/μ-CaCO_3_ colloidal material. The mechanism of μ-CaCO_3_ formation involves the creation of nanometer-sized crystallites at the first stage which later aggregate to form micrometer-sized superstructures. nAg introduced during the co-precipitation process can adsorb on such nanocrystallites due to the electrostatic interactions before their aggregation. In such a way, the embedded nAg stay firmly attached inside the CaCO_3_ matrix and are not prone to fast releasing through the pores of μ-CaCO_3_ (Petrov et al. 2005). SEM analyses have proven that the obtained spherical microparticles are practically monodisperse with the diameter around 2 μm (Fig. 3). This was achieved by the addition of PSS anionic polyelectrolyte and application of ultrasound agitation. To prove the presence of nAg in the nAg/μ-CaCO_3_, the carbonate matrix was dissolved using 0.2 M EDTA and then the UV/Vis spectrum of such solution was acquired (Fig. 4), in which characteristic peak for nAg (420 nm) was observed. This experiment, however, cannot be used to determine the content of silver because dissolution was not complete and Ag^+^ was only partially chelated by EDTA. The total silver content in nanoparticles was estimated by AAS. It was found that about 21 % of silver originally present in solution was successfully embedded into μ-CaCO_3_ matrix, thus making 0.015 % mass of the whole material. Such content of nAg is more than enough to kill bacteria efficiently even if the silver would be present only in the form of Ag^+^ ions (Lok et al. 2006). In addition, EDS analysis of the milled microparticles was performed confirming the presence of embedded nAg in CaCO_3_ matrix (see Supplementary Materials).

**Fig. 5:**
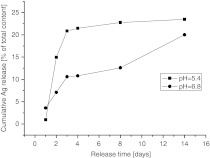
Sustained release of silver into buffer solutions

Sustained release of silver into the buffer solutions was then studied. It has been proven that silver is firmly embedded into microcapsules and can be slowly released over long period of time (Fig. 5). More rapid release of silver in the buffer with lower pH indicates a significant contribution of CaCO_3_ dissolution to the releasing process. The initial ca. 25 % release of silver can be attributed to the presence of the most loosely attached fraction of nanoparticles. Further release of nAg, which are deeply embedded and/or firmly attached to the matrix should be much slower and most likely can occur with disintegration or dissolution of the microparticles (Petrov et al. 2005).

**Fig. 6:**
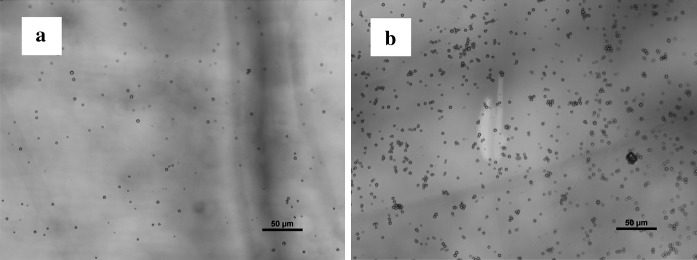
Optical microscopy images of nAg/μ-CaCO_3_ (**a**) and nAg/μ-CaCO_3_ modified with PAH layer (**b**) on mica surface

Surface of nAg/μ-CaCO_3_ microparticles was later modified by the adsorption of positively charged polyelectrolytes like PAH. The modified material, with positive surface charge, was much easier adsorbed on the negatively charged mica than unmodified material as it can be observed in the optical microscopy images (Fig. 6).

**Fig. 7:**
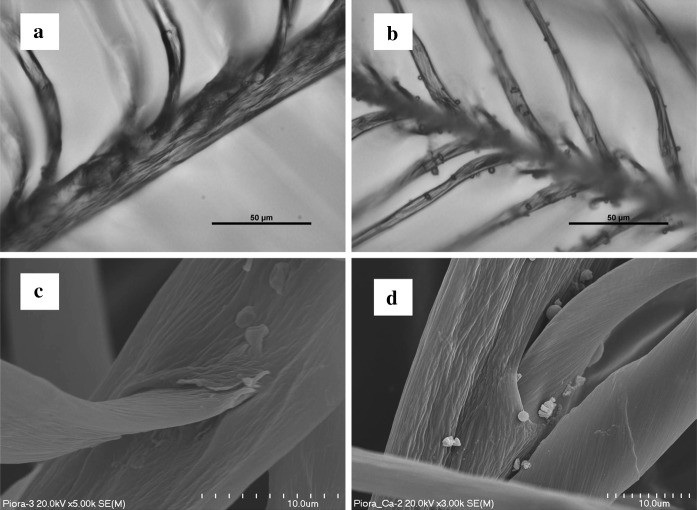
Optical microscopy images of **a** native feathers, **b** feathers covered with nAg/μ-CaCO_3_ and SEM pictures of **c** native feathers, **d** feathers covered with nAg/μ-CaCO_3_

Antimicrobial activity of the nAg/μ-CaCO_3_ microparticles was then tested. High quality down feathers were immersed in nAg/μ-CaCO_3_ aqueous dispersion. Numerous microparticles were attached to feathers successfully, as observed using optical and electron scanning microscopes (Fig. 7). It is worth noting that the microparticles were so firmly attached to the feathers that even high vacuum conditions used during SEM analyses did not remove them.

**Fig. 8:**
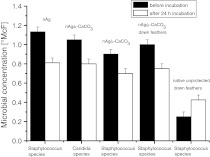
The results of microbial tests for different species. Above each *column*, the antimicrobial additives are listed and below are the names of employed microbial species

The nAg/μ-CaCO_3_ material itself as well as the feathers covered with this agent limited the microbial proliferation. After 24-h incubation, there was a clear decrease in microbial concentration in both cases (Fig. 8). For the comparison, dispersion of sole nAg was used showing similar antimicrobial activity. In contrast, the native unprotected feathers serving as a nutrient accelerated the microbial growth (Fig. 8). Thus, it can be concluded that nAg/μ-CaCO_3_ retains its antimicrobial activity and application of nAg in such a form may limit the contact of the nanoparticles with human body that is of importance considering their long term influence.

## Conclusion

Novel hybrid antibacterial microparticles, nAg/μ-CaCO_3_, consisting of nAg incorporated into calcium carbonate/polymer microparticles (μ-CaCO_3_), were obtained in a simple synthetic procedure carried out in aqueous environment and involving non-toxic compounds. Originally prepared nAg with diameters in the range of 30–50 nm were used in co-precipitation process which results in the formation of nAg/μ-CaCO_3_ spherical microparticles. The obtained material enabled sustained release of nAg, thus limiting the possible environmental problems. Surface charge of the matrix can be readily modified by adsorption of polycations expanding potential applications of nAg/μ-CaCO_3_ to various surfaces. This novel material can be safely stored as a white powder without the risk of losing its antimicrobial activity, in contrast to the sole nAg which tend to aggregate when dried. nAg/μ-CaCO_3_ system was shown to be efficient in limiting the proliferation of microorganisms that makes it suitable for applications involving contact with human skin. As an example, the successful application of nAg/μ-CaCO_3_ for the protection of down feathers against microorganisms was presented.

## Electronic supplementary material

Below is the link to the electronic supplementary material.

Supplementary material 1 (DOC 634 kb)
