# Supplementary material for: Hybrid calcium carbonate/polymer microparticles containing silver nanoparticles as antibacterial agents
Source: J Nanopart Res. 2012 Nov 25;14(12):1313. doi: 10.1007/s11051-012-1313-7 (PMC3517803; doi:10.1007/s11051-012-1313-7)
Supplement: Supplementary file 1 — Supplementary material 1 (DOC 634 kb) [file 11051_2012_1313_MOESM1_ESM.doc]

**Supplementary Materials**

­­­­HYBRID CALCIUM CARBONATE/POLYMER MICROPARTICLES CONTAINING SILVER NANOPARTICLES as antibacterial agentS

Maciej Długosz, Maria Bulwan, Gabriela Kania, Maria Nowakowska*, Szczepan Zapotoczny*

*Jagiellonian University, Faculty of Chemistry, Ingardena 3, 30-060 Krakow, Poland,*

*e-mail:* [zapotocz@chemia.uj.edu.pl](mailto:maciej.dlugosz@uj.edu.pl), tel.: +48 12 6632252, fax: +48 12 6340515

***Synthesis and characterization of enriched nAg/μ-CaCO_3_ material.***

To reach the sensitivity of EDS technique a hybrid material enriched with silver nanoparticles was prepared. To do this native nAg colloid was concentrated by evaporation of water (from the volume of 200 ml to 20 ml) using evaporator and used during the synthesis of the hybrid material as described in the paper. The enriched hybrid material was dried, milled in the agate mortar, deposited on the carbon tape, coated with sputtered carbon and investigated using Hitachi S-4700 s SEM microscope with EDS Noran Vantage system. The pictures of the disintegrated microparticles were taken (Figure S1) and the EDS analysis confirmed that silver is present in the material. Its content was estimated to be 0.97 weight %. (Figure S2). The copper and zinc signals derived from the support on which the sample was mounted prior to the measurement. The sulfur signal comes from the PSS polymer.


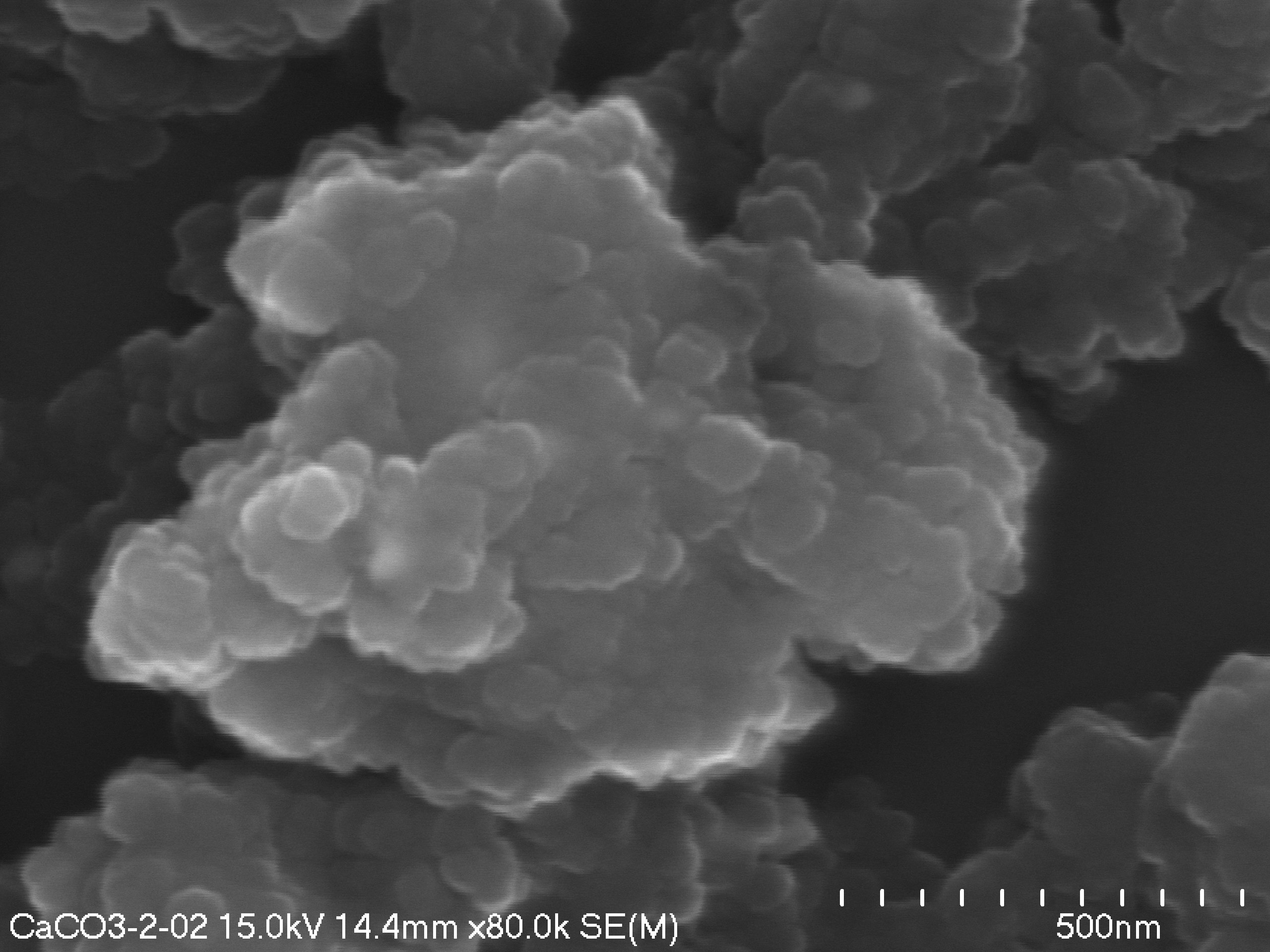


**Figure S1.** SEM image of milled nAg/μ-CaCO_3_ microparticles.


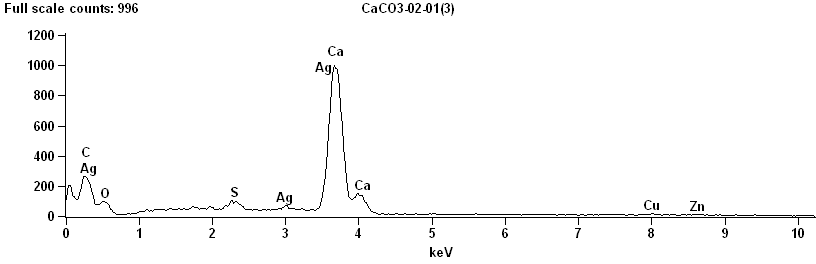


| ***Element***  ***Line*** | ***Net***  ***Counts*** | ***Net Counts***  ***Error*** | ***Weight %*** | ***Weight %***  ***Error*** | ***Atom %*** | ***Atom %***  ***Error*** | ***Formula*** | ***Compnd %*** | ***# Cations*** |
| --- | --- | --- | --- | --- | --- | --- | --- | --- | --- |
| ***C K*** | 2414 | +/- 108 | 56.68 | +/- 2.54 | 71.87 | +/- 3.22 | C | 56.68 | 86.112 |
| ***O K*** | 1089 | +/- 71 | 21.04 | +/- 1.37 | 20.03 | +/- 1.31 | O | 21.04 | --- |
| ***S K*** | 1132 | +/- 106 | 0.72 | +/- 0.07 | 0.34 | +/- 0.03 | S | 0.72 | 0.412 |
| ***Ca K*** | 21958 | +/- 414 | 19.20 | +/- 0.36 | 7.29 | +/- 0.14 | Ca | 19.20 | 8.740 |
| ***Cu K*** | 198 | +/- 61 | 0.69 | +/- 0.21 | 0.17 | +/- 0.05 | Cu | 0.69 | 0.198 |
| ***Zn K*** | 166 | +/- 56 | 0.70 | +/- 0.24 | 0.16 | +/- 0.05 | Zn | 0.70 | 0.195 |
| ***Ag M*** | 784 | +/- 165 | 0.97 | +/- 0.20 | 0.14 | +/- 0.03 | Ag | 0.97 | 0.164 |
| ***Total*** |  |  | 100.00 |  | 100.00 |  |  | 100.00 | 95.821 |

**Figure S2.** EDS analysis of the enhanced material.
